# Implementing blended learning in emergency airway management training: a randomized controlled trial

**DOI:** 10.1186/s12873-018-0152-y

**Published:** 2018-01-15

**Authors:** Madeleine Huei Tze Kho, Keng Sheng Chew, Muhaimin Noor Azhar, Mohd Lotfi Hamzah, Kee Man Chuah, Aida Bustam, Hiang Chuan Chan

**Affiliations:** 1 Emergency Medicine and Trauma Department, Sarawak General Hospital, Jalan Hospital, Kuching, 93586 Sarawak Malaysia; 2 Faculty of Medicine and Health Sciences, Universiti Malaysia Sarawak, Kota Samarahan, 94300 Sarawak Malaysia; 3 Faculty of Medicine, University of Malaya, Kuala Lumpur, 50603 Malaysia; 4 Emergency Medicine and Trauma Department, Hospital Sultanah Nur Zahirah, Kuala Terengganu, 20400 Trengganu Malaysia; 5 Faculty of Language Studies and Communication Studies, Universiti Malaysia Sarawak, Kota Samarahan, 94300 Sarawak Malaysia

**Keywords:** Emergency airway management, Blended learning, Online learning, E-learning

## Abstract

**Background:**

While emergency airway management training is conventionally conducted via face-to-face learning (F2FL) workshops, there are inherent cost, time, place and manpower limitations in running such workshops. Blended learning (BL) refers to the systematic integration of online and face-to-face learning aimed to facilitate complex thinking skills and flexible participation at a reduced financial, time and manpower cost. This study was conducted to evaluate its effectiveness in emergency airway management training.

**Methods:**

A single-center prospective randomised controlled trial involving 30 doctors from Sarawak General Hospital, Malaysia was conducted from September 2016 to February 2017 to compare the effectiveness of BL versus F2FL for emergency airway management training. Participants in the BL arm were given a period of 12 days to go through the online materials in a learning management system while those in the F2FL arm attended a-day of face-to-face lectures (8 h). Participants from both arms then attended a day of hands-on session consisting of simulation skills training with airway manikins. Pre- and post-tests in knowledge and practical skills were administered. E-learning experience and the perception towards BL among participants in the BL arm were also assessed.

**Results:**

Significant improvements in post-test scores as compared to pre-test scores were noted for participants in both BL and F2FL arms for knowledge, practical, and total scores. The degree of increment between the BL group and the F2FL arms for all categories were not significantly different (total scores: 35 marks, inter-quartile range (IQR) 15.0 – 41.0 vs. 31 marks, IQR 24.0 – 41.0, *p* = 0.690; theory scores: 18 marks, IQR 9 – 24 vs. 19 marks, IQR 15 – 20, *p* = 0.992; practical scores: 11 marks, IQR 5 -18 vs. 10 marks, IQR 9 – 20, *p* = 0.461 respectively). The overall perception towards BL was positive.

**Conclusions:**

Blended learning is as effective as face-to-face learning for emergency airway management training of junior doctors, suggesting that blended learning may be a feasible alternative to face-to-face learning for such skill training in emergency departments.

**Trial registration:**

Malaysian National Medical Research NMRR-16-696-30190. Registered 28 April 2016.

**Electronic supplementary material:**

The online version of this article (10.1186/s12873-018-0152-y) contains supplementary material, which is available to authorized users.

## Background

One essential skill every emergency doctor needs to have is the skill of emergency airway management. Conventionally, emergency airway management training is carried out via face-to-face learning workshops. Unfortunately, these workshops can only admit a limited number of participants per course and the participants are required to be there in person throughout the entire workshop. There are inherent cost, time, place and manpower limitations in running such conventional face-to-face workshops. Hence, it is useful to explore the feasibility of using the blended learning approach in overcoming the aforementioned limitations.

Blended learning (BL) refers to the systematic integration of online and face-to-face learning (F2FL) in order to facilitate critical, creative and complex thinking skills [1]. As poignantly alluded by Garrison and Kanuka (2004), while internet and technology is certainly involved, BL is not just about delivering or uploading old contents in a new medium [1]. Rather, it involves a fundamental reconceptualization and restructuring of the entire learning process [1]. In other words, the focus is not on the technological tools [2], but on how best to combine certain aspects for online learning (e.g. topics that require extensive theoretical discussion) and other aspects (e.g. skills training) for F2FL [3]. A well-blended course, therefore, should translate into better learning outcomes at a reduced long-term cost, time and manpower while at the same time enable flexible participation from any location at any time [2, 4]. Essentially, instead of bringing the people to learning, BL is about bringing learning to the people [5] without compromising on the quality of instructional and content delivery.

A closely related learning approach known as the flipped learning (also called “the inverted classroom”), is a pedagogical model which reverses what typically occurs in classes [6]. Students are first exposed to the material outside of class, typically in the form of video-based lectures, and then class time is transformed into an interactive learning environment where students engage in activities for active learning such as problem solving, discussion, and analysis [6]. In this study, BL rather flipped learning was chosen as emergency airway management training still requires the instructor to take a central role during practical skills learning.

In a systematic review on the role of BL in clinical education, Rowe et al. (2012) emphasized that as clinical education is highly context-dependent, generalization across disciplines can be challenging, because the success of implementing BL in one clinical domain does not necessarily mean that it will have similar value in another domain [2]. With that in mind, we embarked on a study with the primary outcome of evaluating the effectiveness of implementing BL in emergency airway management workshop for junior doctors as compared to F2FL. It is hypothesized that BL is at least as effective as F2FL workshop for emergency airway management training. Secondary outcomes of this study are 1) the participants’ subjective perception of BL; and since it has been shown in literature that the lack of information and communication technology (ICT) skills is a concern in BL [4], 2) subgroup analyses of the influence of the participants’ prior ICT skills on their test performance.

## Methods

This was a single center, single blinded, prospective randomised controlled trial (from September 2016 to February 2017) aimed to compare the efficacy of BL versus F2FL in emergency airway management workshop for junior doctors. In the F2FL arm, the participants attended an 8-h one-day F2FL lectures on emergency airway management and discussion followed by a day of hands-on session consisting of simulation skill training with airway manikins.

For the BL arm, course materials consisting of videos, manuals and quizzes were first developed and made available to participants through an online learning management system (openlearning.com). Course contents were standardized with those delivered in the F2FL arm. Social networking services were utilized to enhance discussions among participants and the instructors. Participants were given a period of 12 days (estimated at 40 min per day) to go through the materials during their own personal time, followed by a day of hands-on session consisting of simulation skill training with airway manikins (similar to that in F2FL arm).

Ethical approval was obtained from the Medical Research and Ethics Committee, Ministry Of Health Malaysia and the study was registered under the Malaysian National Medical Research Register (NMRR, website URL: https://tinyurl.com/y9mhztog) with the research number of NMRR1669630190 prior to the commencement of the study.

### Participants

Junior doctors working in the emergency department of Sarawak General Hospital, Malaysia were invited to participate in this study. We define a “junior doctor” as a doctor in his or her second and third year of clinical service. The reason for choosing junior doctors in their second or third year of clinical service (rather than those in their first year of service) is because these doctors have had at least 1 year of basic clinical experience which we believe enables them to better comprehend the contents of this training and to actively participate in the discussions with critical thinking skills. Any junior doctor who had participated in any prior airway training course was excluded from the study. Informed consents were obtained from all participants prior to their participation in this study. The detailed descriptions of the participants are given in Table 1.

**Table 1:** Baseline characteristics of participants

Variable	Face-to-face Group (*N* = 15)	Blended Group (N = 15)
Age (year)		
Median	27	27
Interquartile range	2	2
Sex – n (%)		
Male	8 (53.3)	10 (66.7)
Female	7 (46.7)	5 (33.3)
Years of service – n (%)		
Second	8 (53.3)	10 (66.7)
Third	7 (46.7)	5 (33.3)
Current position – n (%)		
Houseman	8 (53.3)	8 (53.3)
Medical Officer	7 (46.7)	7 (46.7)
Prior related training courses – n (%)		
Basic Life Support	9 (60.0)	8 (53.3)
Neonatal Resuscitation Programme	12 (80.0)	13 (86.7)
Pediatric Life Support	1 (6.7)	1 (6.7)
Advanced Pediatric Life Support (APLS)	0 (0.0)	0 (0.0)
Pediatric Advanced Life Support (PALS)	3 (20.0)	2 (13.3)
Advanced Cardiovascular Life Support (ACLS)	4 (26.7)	4 (26.7)
Advanced Life Support (ALS)	0 (0.0)	2 (13.3)
Advanced Trauma Life Support (ATLS)	1 (6.7)	0 (0.0)
Malaysian Trauma Life Support (MTLS)	0 (0.0)	0 (0.0)
Amount of prior training courses per person - n (%)		
One	3 (20.0)	6 (40.0)
Two	10 (66.7)	4 (26.7)
Three	1 (6.7)	4 (26.7)
Four	1 (6.7)	1 (6.7)

The sample size estimation was calculated based on the 2-mean formula on the G*Power software version 3.1.9.2 [7] based on a study by Lancester et al. in 2012 which compared examination scores of nursing degree students who went through either traditional or blended methods of lecture delivery on pharmacotherapeutics [8]. In that study, it was found that the mean scores were 92.7 +/− 3.8 and 96.6 +/− 1.9 for traditional learning and blended learning respectively [7]. Therefore, using a priori analysis with a 95% power of study with an alpha of 0.05, a total sample size of 28 with 14 in each group was determined. This sample size was further inflated by 10% to compensate for potential drop out, thus making our sample size 30 participants in total, or 15 participants in each arm.

### Materials

Besides training the participants in the fundamentals of airway management, this workshop also aimed to address the safety gaps identified in the 4th National Audit Project of The Royal College of Anaesthetists and The Difficult Airway Society (NAP4). The topics for this workshop were first gleaned, selected and compiled from two existing airway management courses in Malaysia, i.e., the East Coast Airway Course (EAST), as well as the airway management course from a postgraduate emergency medicine training program (i.e., the University of Malaya Emergency Medicine Masters’ Program curriculum). All materials were internally validated via a modified Delphi method to reach a consensus by a panel of experts in emergency medicine (MHTK, KSC, MNA, MLH, AB) with one of them having a special interest in emergency airway management (MLH). These module topics and skill stations were as listed in Additional file 1: Table S1.

### Procedure

The study was divided into two stages. The first stage was the development of teaching contents and assessment questions using a modified Delphi method in three rounds of online discussion. As the experts are based in different locations within Malaysia, a video conferencing discussion was carried as the first round of discussion. In this round, the overarching aims and objectives of the workshop were clarified among the experts. The experts then listed out the probable topics for the workshop as well as the assessment questions. Topics of interest were then emailed by the experts to author MHTK who was responsible in compiling them. In the second round of online discussion, the list of topics were then emailed out by author MHTK to all experts who then scrutinized and gave their suggestions to these topics. The agreed topics were then compiled by author MHTK and emailed out again to all experts for a third round of online discussion. In this round, specific tasks of preparing the lecture notes, presentation slides and video lectures were assigned to specific experts. Experts also contributed the pre-test and post-test assessment questions (comprising of four sections: 1) one best answer (OBA) 2) true/false section (T/F) 3) “fill-in-the-blanks” and 4) practical skills stations) (see Additional file 1: Table S1 for the detailed descriptions). These questions were then vetted and agreed upon by the experts. Any differences in opinion among the experts were resolved via further discussions and consensus.

For participants in the BL arm, the notes were uploaded in the learning management system (URL: https://www.openlearning.com/courses/emergency-airway-management/HomePage); whereas for participants in the F2FL arm, it was in the form of printed handouts). Online activities in the form of quizzes, “fill in the blanks”, and crossword puzzle were created for participants in BL arm. A similar quiz was prepared for those in the F2FL arm as well, but the “fill in the blanks” and crossword puzzle were not administered to the F2FL arm as these are optional enhancing activities in a BL setting.

The learning management system was then checked independently by 2 of the researchers (MNA and KMC); and all errors were rectified before the online course was opened for enrollment. All participants (from both arms of the study) participated in this study without any fee or charges.

The second stage of this study was participant recruitment and randomization as well as the implementation of educational interventions. Junior doctors who consented to this voluntary, anonymous study were first randomized using an online number generator (http://www.graphpad.com/quickcalcs/randMenu/) into either the BL arm or the F2FL arm. Upon registration, all participants completed a pre-test theory & practical assessment by an independent emergency physician who was blinded to the participants’ study arms. To assess their information and communication technology (ICT) skills, participants in the BL arm also completed a validated questionnaire on ICT skills using a Likert scale from 0 to 10 where 0 means the participant finds it the easiest to perform the ICT task and 10 means the participant finds it the hardest to perform the ICT task [9]. The information gleaned from these participants’ prior ICT skills were then analyzed for any possible correlation between their ICT skill proficiency levels with the scores they obtained. Participants from the BL arm had access to the online learning materials, online quizzes and discussions via an online blended learning classroom for 12 days before they joined participants from the F2FL arm for the one-day hands-on session. A social networking messaging application was utilized to enhance discussions among participants and the instructors. For the F2FL arm, the exact same lecture notes were made available to them for 12 days before the first day of the workshop. They then attended an 8-h one-day of F2FL lectures covering all of the above-mentioned modules on emergency airway management using PowerPoint slides with the same content as that which was used for video lectures. Time was allocated after each lecture for discussion and to answer any questions that the participants had. They also participated in a group quiz and also watched video demonstration of certain procedures relevant to emergency airway management (available also on the online learning system). Participants from the F2FL arm underwent a one-day (or 8 h) training consisting of face-to-face classroom lectures. This was followed by a one-day hands-on session consisting of simulation skill training with airway manikins. All participants then completed a post-test theory and practical assessment by the same emergency physician who conducted the pre-test practical assessment. The assessor was blinded to the participants’ arm all throughout the study.

In addition, participants from the BL arm also answered a validated quantitative e-learning experience questionnaire adapted from Ginns & Ellis (2007) [10] and a qualitative questionnaire to gauge their perception towards blended learning, adapted from Larsen (2012) [11]. All the quantitative data that was collected was then analyzed using IBM SPSS Statistics 15.0 for Windows. Thematic analysis was manually performed to code or interpret the qualitative data.

## Results

A total of 30 junior doctors participated in this study (with 15 participants in each of the 2 arms). None of these participants dropped out from the study. The median age of each group was 27 with the interquartile range of 2 years. In terms of years of experience, 8 (53.3%) participants and 10 (66.7%) participants in the F2FL and BL arm respectively had 2 years of clinical experience. The rest of the participants had 3 years of clinical experience. The detailed descriptions of the participants are presented in Table 1. Non-parametric tests were used, as the assumptions of normality were not satisfied for the variables of theory, practical and total scores. When analyzed using Wilcoxon Signed Rank Test, the post-test scores for the theory, practical and total scores were significantly higher than the pre-test for both BL and F2FL arms (see Table 2).

**Table 2:** Comparison of pre-test vs. post-test scores for theory, practical and total scores

	Group	Pre-test	Post-test	Wilcoxon Signed Rank Test
		Median IQR (1st, 3rd)	Median IQR (1st, 3rd)	
Theory score	F2FL	62 (54, 67)	80 (75, 85)	Z = −3.416 *P* = 0.001
	BL	58 (56, 64)	79 (72, 82)	Z = −3.409 *P* = 0.001
Practical score	F2FL	64 (58, 72)	80 (74, 86)	Z = −3.079 *P* = 0.002
	BL	65 (57, 72)	75 (70, 82)	Z = −3.068 *P* = 0.002
Total score	F2FL	124 (119, 131)	156 (151, 164)	Z = −3.409 *P* = 0.001
	BL	124 (113, 136)	155 (144, 162)	*Z* = −3.409 *P* = 0.001

F2FL means participants in face-to-face learning arm; BL means participants in blended learning arm

However, while there were significantly higher scores in all post-tests compared to the pre-tests, the degree of increment was not significantly more in one arm compared to the other. In particular, while the median increment from pre-test to post-test total score was higher in the BL arm than in the F2FL arm; i.e., 35 marks (inter-quartile range, IQR 15.0 - 41.0; mean rank =14.83; sum of ranks 222.50) in the BL arm compared to 31 marks (IQR 24.0 - 41.0; mean rank = 16.17; sum of ranks 242.50) in the F2FL arm, this was not statistically significant (U = 102.5; z = −0.415; *p* = 0.690). For the theory score, the median increment from pre-test to post-test was about the same across both arms; with 18 marks (IQR 9 - 24; mean rank = 14.27; sum of ranks = 232) and 19 marks (IQR = 15 - 20; mean rank = 15.53; sum of ranks = 233) in BL arm and F2FL arm (U = 112; z = −0.21; *p* = 0.992). For the practical score, the median increment from pre-test to post-test was also almost the same across both arms; with 11 marks (IQR 5 -18; mean rank = 15.47; sum of ranks = 214) and 10 marks (IQR = 9 – 20; mean rank = 16.73; sum of ranks = 251) in BL arm and F2FL arm respectively (U = 94; z = −0.769; *p* = 0.461).

For the subgroup analysis of the participants’ responses to BL experience, 12 out of 15 participants (80%) were satisfied with the overall quality of the online materials and activities in the BL (Item no. 32). With regards to the participants’ interaction and engagement in online postings (Items 7, 16, 18 and 21), while the majority of them agreed that reading other participants’ on-line submissions helped to clarify (Item no. 7; 53.3% of the participants), aided in their own “understanding” of some of the contents (Item no. 18; 73.3% of the participants) and appeared to have motivated them to read further (Item no. 21; 60.0% of the participants). However, only 20% of the 15 participants indicated that they had interacted with other students’ on-line postings (Item no. 20). For other detailed responses of the participants’ BL experience, refer Table 3.

**Table 3:** Responses of participants in BL arm on their e-Learning experience

No	Item	Mean*	S.D.*	Likert scale response [N (%)] **			
				Disagree	Neutral	Agree	Missing data
1	To do well in the on-line quizzes all you really need is a good memory.	3.31	0.263	1 (6.7)	2 (13.3)	12 (80.0)	–
2	The teacher used the on-line environment when appropriate to keep students informed about results.	3.77	0.166	0 (0.0)	4 (26.7)	11 (73.3)	–
3	I received too much feedback on-line from my teacher.	2.62	0.180	4 (26.6)	10 (66.6)	0 (0.0)	1
4	The teacher’s responses on-line motivated me to learn more deeply.	3.85	0.191	0 (0.0)	4 (26.6)	10 (66.6)	1
5	The teacher helped to guide on-line discussions between students	4.00	0.160	0 (0.0)	2 (13.3)	13 (86.7)	–
6	The teacher used the on-line environment to regularly update students about relevant unit of study information.	4.08	0.178	0 (0.0)	2 (13.3)	13 (86.7)	–
7	Reading other students’ on-line submissions clarified some of my own ideas.	3.46	0.215	1 (6.7)	6 (40.0)	8 (53.3)	–
8	The on-line teaching materials in this unit of study are extremely good at explaining things.	3.92	0.239	1 (6.7)	2 (13.3)	12 (80.0)	–
9	The teacher’s interaction with me on-line encouraged me to get the most out of my learning.	4.00	0.160	0 (0.0)	2 (13.3)	13 (86.7)	–
10	On-line quizzes helped me to learn effectively.	4.15	0.154	3 (20.0)	4 (26.7)	8 (53.3)	–
11	The workload for the on-line component of this unit of study is too heavy.	2.54	0.268	6 (40.0)	6 (40.0)	3 (20.0)	–
12	The teacher’s on-line responses motivated me to do more on-line learning than I would have done otherwise.	3.85	0.222	0 (0.0)	6 (40.0)	9 (60.0)	–
13	Information needed to understand the purpose and contents of the unit was integrated in one place on-line.	3.77	0.281	2 (13.3)	3 (20.0)	10 (66.7)	–
14	I generally had enough time to understand the things I had to learn on-line.	3.54	0.243	1 (6.7)	6 (40.0)	8 (53.3)	–
15	I didn’t receive enough helpful on-line feedback from my teacher.	2.15	0.249	8 (53.3)	7 (46.7)	0 (0.0)	–
16	I interacted with students’ on-line postings/submissions even if they weren’t assessed.	2.77	0.231	4 (26.7)	8 (53.3)	3 (20.0)	–
17	The on-line activities are designed to get the best out of students.	3.92	0.137	0 (0.0)	3 (20.0)	12 (80.0)	–
18	Other students’ on-line submissions helped me understand my ideas from a new perspective.	3.77	0.166	0 (0.0)	4 (26.7)	11 (73.3)	–
19	The guidelines for using on-line discussions were clear to me.	3.92	0.211	0 (0.0)	4 (26.7)	11 (73.3)	–
20	The on-line teaching materials are designed to really try to make topics interesting to students.	3.92	0.239	1 (6.7)	2 (13.3)	12 (80.0)	–
21	Other students’ on-line submissions encouraged me to investigate further sources of knowledge.	3.69	0.208	0 (0.0)	6 (40.0)	9 (60.0)	–
22	The sheer volume of work for the on-line component of this unit of study means it can’t all be thoroughly comprehended.	2.92	0.348	3 (20.0)	7 (46.7)	5 (33.3)	–
23	The on-line learning materials helped me to learn during the face-to-face situations in this unit of study.	3.62	0.213	1 (6.7)	4 (26.7)	10 (66.7)	–
24	It was clear if on-line resources were related to assessment.	4.23	0.201	0 (0.0)	2 (13.3)	13 (86.7)	–
25	The on-line activities helped me to understand the face-to face activities in this unit of study.	3.85	0.191	0 (0.0)	4 (26.7)	11 (73.3)	–
26	The on-line materials supported some key assessment items in this unit.	4.08	0.178	0 (0.0)	1 (6.7)	14 (93.3)	–
27	The relationship between the on-line resources and the whole unit of study was clarified on the unit’s website.	3.69	0.237	1 (6.7)	5 (33.3)	9 (60.0)	–
28	The teacher helped to focus on-line discussions between students.	4.00	0.196	0 (0.0)	3 (20.0)	12 (80.0)	–
29	Information needed for assignments was integrated in the one place on-line.	3.85	0.222	1 (6.7)	3 (20.0)	11 (73.3)	–
30	It was clear to me how the website for this unit related to the whole unit of study	3.92	0.239	1 (6.7)	3 (20.0)	11 (73.3)	–
31	The teacher ensured continuous access to the relevant on-line materials throughout the semester.	4.08	0.239	0 (0.0)	4 (26.7)	11 (73.3)	–
32	Overall, I was satisfied with the quality of the on-line materials and activities of this unit of study.	4.15	0.274	1 (6.7)	2 (13.3)	12 (80)	–

items 3, 4, 5, 9, 12, 15 and 28 represent “quality of teaching in e-learning context”

items 7, 16, 18, 21 = “student interaction and engagement”

items 13, 19, 29 = “clarity of goals and standards for online component”

items 8, 17, 20, 23 = “quality of online resources”

items 1, 10 and 26 = “appropriateness of assessment in e-learning context”

items 11, 14 and 22 = “appropriateness of workload related to online materials & activities”

items 2, 6 and 31 = “issues related to student management”

items 24, 25, 27 and 30 = “degree to which online materials and activities support face-to-face learning”

item 32 = “overall satisfaction with the quality of online materials and activities”

In terms of the level of prior ICT skills of the participants, it was found that most participants did not consider the list of ICT skills in the questionnaire to be difficult, as all items or tasks listed in the questionnaire had responses concentrated in the easy category (> 60.0%). Less than 10% of the participants considered any of the items as difficult (see Additional file 1: Table S2 for the detailed responses). Spearman’s correlation was used to determine the correlation between prior ICT skills of the participants and their e-learning experience. There was no significant correlation between these two variables (r_s_ = −0.411, *n* = 15, *p* = 0.128). In other words, the participants’ e-learning experience did not seem to be affected by the level of their ICT skills. Besides that, Spearman’s correlation was also used to determine the correlation between the ICT skills of the participants and the BL course outcome in terms of the increment of score from pre-test to post-test. It was found that there was no significant correlation between the level of prior ICT skills of participants and the blended learning course outcome (increment of total score among participants in BL group) (r_s_ = −0.64, *n* = 15, *p* = 0.822).

From the qualitative questionnaire on the participants’ perspectives towards BL, it was noted that most of the participants responded positively to BL (refer Table 4 for all highlighted responses). For example, when asked to describe the training they received via blended learning (Question 3), two participants mentioned that BL affords the learning flexibility allowing them to go through the materials *“over and over again”.* The course was also described as *“informative”* (4 responses), *“comprehensive”* (1 response), *“concise”* (1 response), *“efficient”* (1 response), *“enjoyable”* (1 response), *“well-organised”* (1 response), *“good”* (3 responses), *“ample time provided”* (1 response), and *“extremely good and valuable training which is hard to learn via textbook only”* (1 response). One person however, remarked that *“learning through the blended program was a little boring”* as the videos were monotonous*.* In response to the question (question no. 12) on what would improve the participants’ involvement in online discussions, one participant mentioned that the online facilitators/instructors should have taken a more active role to pose more questions to the participants because when the participants were not asking questions, it could be because *“we don’t know what we don’t know”.*

**Table 4:** Qualitative Questionnaire on Participants’ Perspective towards Blended Learning Emergency Airway Management Training

Questions	Highlighted Responses
Why did you take part in this program (emergency airway management training)?	To improve one’s knowledge (12 out of 15 participants) and skills (5 responses)
Would you recommend this course (blended learning emergency airway management training) to a friend? Why?	All participants responded “yes” to recommend this course to a friend because it was beneficial (9 responses), informative (9 responses), simple and concise (2 responses), and helped to improve skills (6 responses) and the videos are accessible anytime and at any location (1 response).
How would you describe the training you received via blended learning?	“Informative” (4 responses), “comprehensive” (1 response), “concise” (1 response), “efficient” (1 response), “enjoyable” (1 response), “well-organized” (1 response), “good” (3 responses), “ample time provided” (1 response), “extremely good and valuable training which is hard to learn via textbook only” (1 response), “affords flexibility in learning” (2 responses), “can go through the materials over and again” (1 response), “a little boring” (1 response).
Did you do all the activities in the course? Why or why not?	Yes (13 responses). No (2 responses) because “it was getting boring” (1 response)
Do you feel this course has any advantage(s) for the students? Which?	Yes (15 responses); all of the modules (1 response), airway anatomy (1 response), algorithms (1 response), pharmacology (1 response), difficult intubation (1 response) and rapid sequence induction (1 response).
Do you feel this course has any disadvantages for the students? Which?	Yes (4 responses), as it requires discipline (2 responses), good internet connection (1 response) and more engaging videos as “monotonous presentation had me losing my concentration” (1 response).
In which class, do you think you would work more actively: in a face-to-face classroom or in an online learning? Why?	Online learning (4 responses) because it affords flexibility (2 responses). Face-to-face classroom (6 responses) it is “not easily distracted” in face-to-face classroom (1 response), and it allows for “interaction and discussion there and then” (1 response), and that they would know better what mistakes were made in face-to-face classroom (1 response).
What did you like the most about this course?	Materials and knowledge gained (8 responses) as there “depths in the knowledge and skill that I can acquire regarding airway management” (1 response) and “easy learning with good teaching and guidance” (1 response), Flexibility of time (2 responses), “making it easy to accommodate in a busy schedule” (1 response).
Would you like to take more courses that use blended learning? Why?	Yes (14 responses), because of the flexibility of time and place (3 responses), “being able to re-play the videos to comprehend the important points that I might have missed” (1 response), ability to monitor own progress (1 response), and “for knowledge and self-improvement” (2 responses). No, still prefer traditional lecture-style of learning (1 response).
If you could suggest changes to this course what would you suggest?	Should allocate longer duration for the course so that they would not have to “cram things daily” (1 response), using better quality audio system in the videos (1 response), to put up more interesting videos (1 response).
What would improve your participation in online discussions?	Incentive for active participation (e.g. achievement points or participation rewards) (1 response), “more involvement by all participants, with everyone taking the initiative to discuss the topics” (1 response) and facilitators/instructors should have taken a more active role to pose more questions to the participants because when the participants were not asking questions, it is because “we don’t know what we don’t know” (1 response).

## Discussion

This study seems to suggest that BL is not inferior to, but as effective as F2FL in emergency airway management training as evidenced by the fact that there is a significant improvement from pre-test to post-test for theory, practical and total scores in the BL arm as well as in the F2FL, but there is no statistically significant difference in terms of the degree of increment of the scores across the two arms. This finding suggests that blended learning can be a feasible alternative to deliver skill-based training materials in a more accessible and affordable manner. The relevance of BL in such emergency medical trainings has also been suggested in other literature [12, 13]. BL has also been shown to be an effective method of training in other medical fields with high levels of student satisfaction [14–16]. A subgroup analysis in this study also suggests that the participants’ prior information and communication technology (ICT) skills do not seem to influence their test performance. This seems to suggest that the chosen BL platform is friendly enough to be learned without much technical difficulty, which in turn promotes a positive virtual leaning environment.

Nonetheless, while the implementation of BL as well as the perception of the participants towards BL were apparently positive, these were not be overly so. One might argue that this was because some (6 out of 15) of the participants in the BL arm seemed to prefer to work in a F2FL environment. For example, some participants mentioned that they would prefer to have interaction and discussions with the instructors and with other participants *“there and then”,* so that they would know better what mistakes they might have made. This is known as the theory of instructor immediacy which describes the ability and availability of the instructor to engage in nonverbal and verbal communications with the learners to enhance their learning experience and to increase their learning motivation [17, 18].

Tobin (1998) described a 3-dimensional framework to evaluate participants’ perception of an online environment with each dimension covering a number of categories [19]. These three dimensions are 1) “emancipatory activities” dimension (the degree of autonomy afforded to the participants in controlling the pace, place and depth of learning) 2) “co-participatory activities” dimension (the collaboration and interaction among participants and with the instructors) and 3) the “qualia” dimension (the degree of satisfaction, enjoyment as well as frustration of the participants with the online learning). In this study, while the dimension of emancipatory activities seems to be rated rather highly by the participants, the dimension of “co-participatory activities” seems to leave much to be desired. For example, a number of participants felt that the instructors should have played a much more active role in the online community by facilitating more active discussions. As Ellaway and Masters (2008) said, instructors should not be the “absent landlord” [20] but rather, be actively guiding the participants.

The limitations of this study include the small sample size as well as the fact that this study only involved participants from a single center. Only internal validation of the contents has been performed for the contents of this workshop (with no external validation yet when the study was carried out). Combining participants from both arms for the one-day practical session could have been a source of confounder, obscuring any potential differences in outcomes between the two interventions. Furthermore, there was no real patient-based outcome measured in the assessment of this study (e.g. the competency in managing emergency airway in real patients). Furthermore, this emergency airway management training only entails contents equivalent of a 2-day F2FL workshop. As such, its effectiveness may not be generalizable to more extensive or lengthy courses. Lastly, while the participants in the BL arm had been instructed not to reveal their course materials to their peers assigned to the F2FL arm and vice versa, the risk of cross-contamination remains. Similarly, participants in both arms were combined together during the practical training session and their interaction and discussions might have a confounding effect on the post-test performance.

Future works that could be performed include embarking on developing BL with more technologically-enabled engaging contents. The SAMR model [21, 22] describes four levels of technology integration and engagement in instructional design. These four levels are S = substitution, A = augmentation, M = modification, and R = redefinition. In the first two stages, technology is merely used as an enhancement tool. In the latter two stages, technology is actually used to transform education. In our BL arm, the face-to-face lectures were substituted by video lectures. This was further augmented by utilization of online quizzes and activities such as crossword puzzles and ‘fill in the blanks’ with instant feedback in the form of answers to help solidify the lessons learnt. One of the ways to advance to “M” or “R” stages of the SAMR model is to create simulation games based on experiential learning theory, flow theory and game design [23], with the aim to further engage students with serious games consisting of challenges appropriate to the skill level that affords immediate feedback. This will likely to result in intense focus and deep learning with high levels of self-satisfaction. Besides that, the effectiveness of BL should also be evaluated on all the four levels of the Kirkpatrick Training Evaluation Model [24]. Based on the Kirkpatrick Training Evaluation Model, we only evaluated Level 1 (reaction towards training) and Level 2 (the degree to which participants acquire the intended knowledge, skills, attitude, confidence and commitment based on their participation in the training). It will be good if further studies can evaluate Level 3 and 4 of Kirkpatrick Training Evaluation Model, i.e., 1) the behaviour change (the degree to which participants apply what they learned during training when they are back on the job) and 2) results (the degree to which the targeted outcomes occur as a result of the training), respectively.

## Conclusion

In conclusion, this study suggests that BL may be a feasible alternative to F2FL for skill-based training such as the emergency airway management training as it was shown that the effectiveness of the BL is non-inferior to F2FL.

## Additional file

Additional file 1: **Table S1** Module Topics, Skill Stations and Assessment Questions in the Emergency Airway Management Workshop and **Table S2** Responses of participants in the blended learning arm on ICT skills. (DOCX 19 kb)

## Abbreviations

BL: Blended learning
EAST: East Coast Airway Course
F2FL: Face-to-face learning
ICT: Information and communication technology
SAMR: Substitution, Augmentation, Modification, and Redefinition model
