# Supplementary material for: Implementing blended learning in emergency airway management training: a randomized controlled trial
Source: BMC Emerg Med. 2018 Jan 15;18:1. doi: 10.1186/s12873-018-0152-y (PMC5769207; doi:10.1186/s12873-018-0152-y)
Supplement: Additional file 1: — Table S1 Module Topics, Skill Stations and Assessment Questions in the Emergency Airway Management Workshop and Table S2 Responses of participants in the blended learning arm on ICT skills. (DOCX 19 kb) [file 12873_2018_152_MOESM1_ESM.docx]

**Additional file 1: Table S1 The List of Module Topics, Skill Stations and Assessment Questions in the Emergency Airway Management Workshop**

| Module topics | | | | Source |
| --- | --- | --- | --- | --- |
| Introduction | | | | EAST |
| Airway Anatomy | | | | UM |
| Airway Assessment | | | | UM |
| Airway Algorithm | | | | EAST |
| Basic Airway Management | | | | EAST |
| Rapid sequence intubation | | | | UM |
| Pharmacology of RSI | | | | UM |
| Advanced Airway | | | | EAST |
| Rescue Techniques | | | | EAST |
| Surgical Airway | | | | EAST |
| Airway Management in Special Circumstances | | | | UM |
|  | | | |  |
| Skills Stations Identified | | | |  |
| Station 1 Rapid sequence intubation | | | | EAST |
| Station 2 Advanced Airway (fiberoptic intubation, video laryngoscope, Bonfils) | | | | EAST |
| Station 3 Airway Algorithm | | | | EAST |
| Station 4 Surgical Airway | | | | EAST |
| Station 5 Airway Megacode | | | | EAST |
|  | | | |  |
| Distribution of the Assessment Questions | | | |  |
| Module | Number of OBA Questions | Number of Multiple True/False Questions | Number of “Fill in the blanks” Questions | Total |
| Introduction | 3 | 3 |  | 6 |
| Airway Anatomy | 5 | 2 | 10 | 17 |
| Airway Assessment | 3 | 2 |  | 5 |
| Airway Algorithm | 4 | 2 |  | 6 |
| Basic Airway Management | 2 | 4 |  | 6 |
| RSI | 4 | 2 |  | 6 |
| Pharmacology of RSI | 2 | 3 |  | 5 |
| Advanced Airway | 1 | 1 |  | 2 |
| Rescue Techniques | 1 | 1 |  | 2 |
| Surgical Airway | 2 | 1 |  | 3 |
| Airway Management in Special Circumstances | 1 | 2 |  | 3 |
| Total | 28 | 23 | 10 | 61 |

*Note:* *UM = Universiti Malaya Emergency Medicine Masters’ Programme Curriculum; EAST = East Coast Airway Course; OBA = one best answer; T/F, true/false*

**Additional file 1: Table S2 Responses of participants in the BL arm on ICT skills**

|  |  |  |  | **Likert scale response [N = 15(%)]** | | |  |
| --- | --- | --- | --- | --- | --- | --- | --- |
| **No** | **Item** | **Mean** | **S.D.** | **0 – 3**  **“Easy”** | **4 – 6 “Neutral”** | **7 – 10 “Hard”** | **Missing data** |
| 1 | Use a word editor to write texts | 2.13 | 1.642 | 12 (80.0) | 3 (20.0) | 0 (0.0) | 0 |
| 2 | Print a document | 1.73 | 1.280 | 13 (86.7) | 2 (6.7) | 0 (0.0) | 0 |
| 3 | Use the computer to organize information | 3.00 | 2.070 | 10 (66.7) | 4 (26.7) | 1 (6.7) | 0 |
| 4 | Start a computer software | 3.40 | 2.414 | 9 (60.0) | 5 (33.3) | 1 (6.7) | 0 |
| 5 | Delete files when they are no longer needed | 1.60 | 1.121 | 14 (93.3) | 1 (6.7) | 0 (0.0) | 0 |
| 6 | Copy a file | 1.67 | 1.175 | 14 (93.3) | 1 (6.7) | 0 (0.0) | 0 |
| 7 | Understand words and terms related to the use of internet | 3.13 | 2.416 | 9 (60.0) | 5 (33.3) | 1 (6.7) | 0 |
| 8 | Edit a file information | 3.13 | 2.232 | 10 (66.7) | 4 (26.7) | 1 (6.7) | 0 |
| 9 | Use the internet to fetch data | 2.47 | 2.356 | 11 (73.3) | 3 (20.0) | 1 (6.7) | 0 |
| 10 | Ask question during a chat | 2.67 | 2.582 | 11 (73.3) | 3 (20.0) | 1 (6.7) | 0 |
| 11 | Delete e-mail | 1.53 | 1.125 | 14 (93.3) | 1 (6.7) | 0 (0.0) | 0 |
| 12 | Print e-mail | 2.07 | 2.219 | 13 (86.7) | 1 (6.7) | 1 (6.7) | 0 |
| 13 | Send e-mail | 1.53 | 1.125 | 14 (93.3) | 1 (6.7) | 0 (0.0) | 0 |
| 14 | Edit text before forwarding it | 2.07 | 1.438 | 13 (86.7) | 2 (13.3) | 0 (0.0) | 0 |
| 15 | Attach a file to an e-mail message | 1.73 | 1.280 | 13 (86.7) | 2 (13.3) | 0 (0.0) | 0 |
| 16 | Find an e-mail from a particular sender or with a specific subject | 1.93 | 1.438 | 13 (86.7) | 2 (13.3) | 0 (0.0) | 0 |
| 17 | Save messages to a file | 2.64 | 2.373 | 10 (71.4) | 3 (21.4) | 1 (7.1) | 1 |
| 18 | Change the password on websites | 2.00 | 1.512 | 12 (80.0) | 3 (20.0) | 0 (0.0) | 0 |
| 19 | Communicate in chat rooms | 1.87 | 1.302 | 13 (86.7) | 2 (13.3) | 0 (0.0) | 0 |
| 20 | Download a new software from the internet | 2.33 | 1.799 | 11 (73.3) | 4 (26.7) | 0 (0.0) | 0 |
| 21 | Search certain terms or issues on the internet using a search engine tool | 2.27 | 1.624 | 12 (80.0) | 3 (20.0) | 0 (0.0) | 0 |
| 22 | Use advanced search parameters as logical operators (and, or, not), filters, etc. | 2.47 | 1.642 | 11 (73.3) | 4 (26.7) | 0 (0.0) | 0 |
| 23 | Communicate through forums (virtual  communities of discussion) on a subject of your interest | 3.07 | 1.831 | 10 (66.7) | 4 (26.7) | 1 (6.7) | 0 |
| 24 | Talk to more than one person using the same screen | 2.67 | 2.257 | 12 (80.0) | 2 (13.3) | 1 (6.7) | 0 |
| 25 | Chat on the internet through audio (sound) | 3.13 | 2.416 | 10 (66.7) | 4 (26.7) | 1 (6.7) | 0 |
| 26 | Chat on the internet through image and sound (webcam or video) | 2.80 | 2.336 | 11 (73.3) | 3 (20.0) | 1 (6.7) | 0 |
| 27 | Send files to other people in a virtual learning environment | 2.47 | 1.959 | 11 (73.3) | 3 (20.0) | 1 (6.7) | 0 |
| 28 | Create a list of favorite pages for easy access | 2.47 | 1.807 | 10 (66.7) | 5 (33.3) | 0 (0.0) | 0 |
| 29 | Find hyperlinks on web pages | 3.36 | 2.136 | 7 (50.0) | 6 (42.9) | 1 (7.1) | 1 |
| 30 | Install plugins (Flash, Shockwave, Java, etc.) required to access documents, photos, videos, webpages | 2.87 | 1.506 | 11 (73.3) | 4 (26.7) | 0 (0.0) | 0 |
| 31 | Access webpages using the standard navigation features (forward, back, home, reload buttons) | 2.87 | 2.200 | 10 (66.7) | 4 (26.7) | 1 (6.7) | 0 |
| 32 | Save files downloaded from the internet at specific locations in the computer’s hard drive | 2.00 | 1.512 | 12 (80.0) | 3 (20.0) | 0 (0.0) | 0 |
| 33 | Compress and decompress files using specific software such as WinZip, WinRAR and others | 2.93 | 2.052 | 11 (73.3) | 3 (20.0) | 0 (0.0) | 0 |
| 34 | Configure audio options on your computer, enabling and disabling the sound as needed | 2.60 | 2.028 | 12 (80.0) | 2 (13.3) | 1 (6.7) | 0 |
| 35 | Evaluate which program is needed for opening files of video and audio (avi, mpeg, mp3, etc.) | 1.93 | 1.223 | 14 (93.3) | 1 (6.7) | 0 (0.0) | 0 |
| 36 | Install software | 2.20 | 1.373 | 13 (86.7) | 2 (13.3) | 0 (0.0) | 0 |
| 37 | Convert text files from one extension to another | 2.87 | 2.167 | 10 (66.7) | 4 (26.7) | 1 (6.7) | 0 |
